# Criticality-Driven Evolution of Adaptable Morphologies of Voxel-Based Soft-Robots

**DOI:** 10.3389/frobt.2021.673156

**Published:** 2021-06-17

**Authors:** Jacopo Talamini, Eric Medvet, Stefano Nichele

**Affiliations:** ^1^ Evolutionary Robotics and Artificial Life Lab, Department of Engineering and Architecture, University of Trieste, Trieste, Italy; ^2^ Department of Computer Science, Artificial Intelligence Lab, Oslo Metropolitan University, Oslo, Norway; ^3^ Department of Holistic Systems, Simula Metropolitan Center for Digital Engineering, Oslo, Norway

**Keywords:** reservoir computing, voxel-based soft robot, evolutionary robotics, criticality, adaptability

## Abstract

The paradigm of voxel-based soft robots has allowed to shift the complexity from the control algorithm to the robot morphology itself. The bodies of voxel-based soft robots are extremely versatile and more adaptable than the one of traditional robots, since they consist of many simple components that can be freely assembled. Nonetheless, it is still not clear which are the factors responsible for the adaptability of the morphology, which we define as the ability to cope with tasks requiring different skills. In this work, we propose a task-agnostic approach for automatically designing adaptable soft robotic morphologies in simulation, based on the concept of criticality. Criticality is a property belonging to dynamical systems close to a phase transition between the ordered and the chaotic regime. Our hypotheses are that 1) morphologies can be optimized for exhibiting critical dynamics and 2) robots with those morphologies are not worse, on a set of different tasks, than robots with handcrafted morphologies. We introduce a measure of criticality in the context of voxel-based soft robots which is based on the concept of avalanche analysis, often used to assess criticality in biological and artificial neural networks. We let the robot morphologies evolve toward criticality by measuring how close is their avalanche distribution to a power law distribution. We then validate the impact of this approach on the actual adaptability by measuring the resulting robots performance on three different tasks designed to require different skills. The validation results confirm that criticality is indeed a good indicator for the adaptability of a soft robotic morphology, and therefore a promising approach for guiding the design of more adaptive voxel-based soft robots.

## 1 Introduction

Traditionally, engineers have designed robotic systems modeled by connected joints made of rigid materials. These rigid-body robots can be programmed to efficiently perform a single task in a predictable way, but often with limited adaptability (Rus and Tolley, 2015).

Soft robots, on the contrary, are designed using soft materials, in order to mimic nature in the way they interact with the environment. Since they are made of soft materials, soft robots are provided with almost infinite degrees of freedom and thus are capable of more natural movements. This allows a variety of different behaviors that were not possible with traditional robots and many new opportunities for robotics, enabled by their greater adaptability (Lipson, 2014). Soft robotic bodies are able to bend and twist with high curvatures, through deforming part of their body in a continuous way (Mazzolai et al., 2012), and thus can move in confined spaces. Since they can adapt their body shape to the environment, soft robots are thus able to manipulate objects, move on rough terrain, and execute rapid and agile manoeuvres underwater. However, due to the intrinsic complexity of their bodies, the design and control of soft robots is a challenging task, which suggests that traditional robotics techniques might not be effective.

Among the existing categories of soft robots, Voxel-based Soft Robots (VSRs) are made of many elastic blocks called voxels, defined by mechanical properties similar to those of biological tissues, which allow them to expand or contract when controlled by an external signal. A VSR is defined by a body (or *morphology*), which is simply an aggregate of voxels, and a brain, which is the control algorithm responsible for actuating its body. Biological inspired meta-heuristics such as Evolutionary Computation (EC) have been extensively applied to the domain of VSRs (Hiller and Lipson, 2011; Cheney et al., 2013; Cheney et al., 2015; Talamini et al., 2019; Medvet et al., 2020a; Ferigo et al., 2021) and have been shown to be a promising approach for both the design and control of VSRs. However the optimization of these robots is often oriented toward a specific task, e.g., a locomotion task, which provides no guarantee on the effectiveness of the resulting designs when subjected to a different task.

In this work, we explore the possibility of automatically designing adaptable soft-robotic bodies by means of EC, such that the resulting bodies are able to successfully accomplish tasks requiring different motor skills. A simple method to find such bodies might consist in evaluating each candidate solution on *all* the relevant tasks, and optimizing the body toward the maximization of the overall performance. However, this approach does not necessarily scale well, since as more and more tasks are to be achieved the computation required to evaluate the robot performances on all tasks may become not practical. In addition, not all the possible tasks are necessarily known in advance, and the definition of a new task would make the results of the previous optimization process outdated.

A better approach may be to identify measures for soft-robot bodies that may evaluate the potential richness of robot dynamics (the potentially available robot behaviors), such that different controllers may be able to successfully operate the robot on different tasks. We propose here a task-agnostic approach for automatically designing adaptable bodies without requiring any information on the tasks, and instead based on the definition of criticality (Bak et al., 1988).

A system in the critical state operates at the edge between two qualitatively different types of behavior. Below the critical state a system is said to be in a subcritical state, where the behavior is highly ordered (static or oscillating between very few distinct states). On the other hand, a system in a supercritical state shows chaotic behavior (unpredictable). At a phase transition between such regimes, a systems is typically able to efficiently respond to a wide range of inputs. Langton (1990) has shown that Cellular Automata at the transition phase, i.e., edge of chaos, are able to efficiently transmit, store, and modify information, and therefore are able to perform complex computations. Biological neural networks and the brain are examples of systems that operate at criticality (Shew and Plenz, 2013; Hesse and Gross, 2014; Heiney et al., 2019). Criticality is typically measured by looking at how activity propagates through the system, such as spike propagation in neural networks. When a certain activity propagates through the system, this is referred to as avalanche. Avalanches may have different size and duration. When the avalanche distribution follows a power law, the system is said to be at criticality. A recent review on criticality and connectivity in relation to neural computation is available at (Heiney et al., 2021).

A popular machine learning approach which relies on dynamical systems computation is Reservoir Computing (RC) (Jaeger, 2001; Maass et al., 2002). In RC, the dynamical system is able to represent input signals in a high-dimensional temporal state, which can be easily distinguished by a linear trained readout. While RC has been implemented in several physical dynamical systems (see Konkoli et al. (2018) and Tanaka et al. (2019) for a recent review), including non-modular soft robots (Li et al., 2012; Nakajima et al., 2013) and origami robots (Bhovad and Li, 2021), it has been shown that the reservoir performs best if the dynamic regime is in a critical state (Schrauwen et al., 2007; Legenstein and Maass, 2007). A VSR body is a dynamical system and may therefore be considered a reservoir computer.

Our hypothesis is that VSR bodies engineered or optimized to perform robustly for a given task may show sub-critical behavior due to their specific topology, i.e., a fairly static or oscillating behavior that works particularly well for one single task. In other words, the required computation is strongly embedded in the body morphology. However, such robust behavior and topology is not particularly adaptive to other tasks requiring very different motor skills and consequently a radically different dynamical behavior. On the other hand, a topology supporting a critical behavior, i.e., a wide range of controllable dynamics, may achieve sub-optimal performances on a wide variety of tasks requiring different motor skills, while not being explicitly optimized for any of them.

In this work we provide a criticality score value, based on the fitting of the empirical avalanches distribution (Bak et al., 1988), coming from the assessment of a body, with a target distribution. Given this score, we evolve VSR morphologies that are optimized for criticality, rather than for a single or a few tasks. The morphology evolution is hence task-agnostic.

**FIGURE 1:**
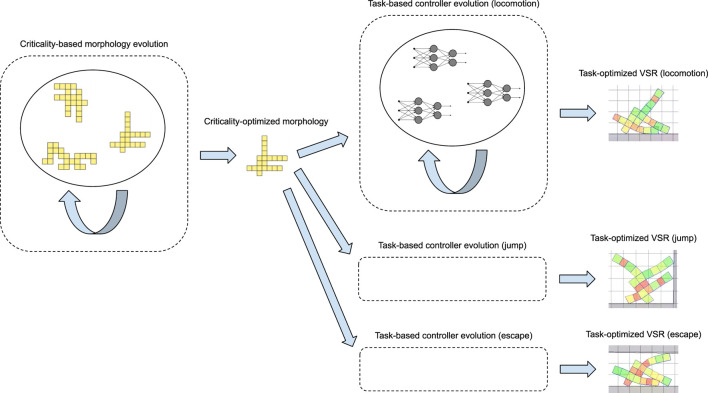
Schematic view of our approach and its experimental evaluation. We first evolve a morphology driving the optimization with a measure of its criticality: during this evolution (left), candidate morphologies are not evaluated on any specific task. Then, we take a criticality-optimized morphology and test its adaptability, i.e., whether a VSR with a task-optimized controller working with this morphology obtains reasonable performance over a set of different tasks. For this evaluation, we evolve a controller (e.g., a neural network) for the criticality-optimized morphology: during each one of these evolutions (right), candidate controllers are evaluated, embedded in a robot with the criticality-optimized morphology, on a specific task.

To validate the adaptability of the morphologies resulting from the evolution, we design three reasonably different tasks: a locomotion task on a plain ground, a jump task, and a task of escape from a narrow cave-like environment, and we test the bodies on these tasks by optimizing their controller on each task. Figure 1 shows an overview of our approach and its experimental evaluation. While our focus is on the adaptability of the VSR morphologies to different tasks based on their criticality, we also investigate whether morphologies evolved with high criticality show adaptability to different controller types, e.g., phase controllers and neural network controllers. Brain adaptability for robotics is a rather consolidated researched topic where adaptive mechanisms may act on short time scales, i.e., through learning. Mechanisms of body adaptation are less understood both for robots and biological organisms. In addition to the bodies evolved toward criticality, we have considered several other bodies for the validation process, some of them inspired by previous works such as Talamini et al. (2019), while others automatically generated thorough algorithms based on randomness. We compare all the robots on the different tasks, and we draw an overall ranking that shows that criticality is indeed a good predictor of the adaptability of a robot morphology. All the experimental work is performed in simulation.

In summary, we experimentally tested the following hypothesis, here formulated as research questions:

RQ1. Do morphologies evolved for criticality obtain a higher criticality value than handcrafted morphologies?
RQ2. Do VSRs with morphologies evolved for criticality obtain an average performance that is not inferior (on a set of different tasks) than that of VSRs with handcrafted morphologies? In both cases, the controllers are evolved for each specific task.
RQ3. Given a pseudo-randomized method for generating morphologies that resemble the ones evolved for criticality, do VSRs with those pseudo-randomized morphologies obtain an average performance that is not inferior (on a set of different tasks) than that of VSRs with handcrafted morphologies? In both cases, the controllers are evolved for each specific task.

Our main findings are the following. 1) Criticality is a good predictor of the potential adaptability of a robot morphology. 2) Criticality as task-agnostic fitness score for the evolution of robot bodies result in the design of robots that are among the most adaptable. 3) Bodies evolved for a specific tasks exhibit almost no adaptability and have indeed particularly low levels of criticality. 4) Morphologies evolved toward criticality show a rather randomized structure that can be mimicked in a random generation procedure for morphologies. Such procedure generate morphologies that are sometimes adaptable and with criticality values that are in a range between those evolved for criticality and those evolved for a specific task. The random generation procedure is computationally fast as compared to EC. 5) The morphologies evolved for criticality may be controlled by different controller types, i.e., phase controller and neural network controller. This suggests that morphologies supporting critical behavior are agnostic to the type of controller being used.

## 2 Background and Related Work

In this section, we first describe voxel-based soft robots. Subsequently, we introduce the concepts related to reservoir computing, a method that relies on dynamical systems for efficient computation. We then provide links between VSRs and RC and finally introduce a beneficial property for dynamical systems and their computational power, namely criticality.

### 2.1 Voxel-Based Soft Robots

A Voxel-based Soft Robots (VSR) is an aggregation of soft cubic blocks, *voxels*, that can vary their volume in response to a control signal. Hiller and Lipson (2012) first introduced VSRs along with a procedure to physically build them using a deformable foam: the control signal corresponded to the atmospheric pressure and voxels were not controllable individually. Later approaches to the realization of VSRs explored different paths as, e.g., silicone cubes actuated by injected air pressure (Kriegman et al., 2020b; Sui et al., 2020) or living matter (Kriegman et al., 2020a). Regardless of the way they are built, VSRs are often designed automatically by means of optimization and simulation: many different designs are simulated and their ability to perform a given task is measured.

In this work, we consider a 2-D variant of simulated VSRs that has been introduced by Medvet et al. (2020b) together with a time-discrete simulation engine that facilitates their optimization. A detailed description of the mechanical model of the VSRs and how they are simulated is given in the cited work and in (Medvet et al., 2020c): we here summarize the salient concepts.

A VSR is defined by its *morphology* and its *controller*. The former describes how the voxels are arranged. The controller determines how the area of each voxel varies over the time, possibly based on the readings of the sensors the VSR is equipped with. The ability of sensing both itself and the environment makes VSRs potentially more effective in performing tasks where perception is beneficial as, e.g., locomotion on uneven terrains (Talamini et al., 2019).

#### 2.1.1 Morphology

A *morphology* is a 2-D grid of voxels: adjacent voxels are rigidly connected at their vertices. In the simulation, the voxel is modeled as a compound of spring-dampers systems, masses, and distance constraints. By varying the parameters of those components, different materials for the voxels can be simulated: in this work, however, we use the same default values for each voxel and hence assume that the voxels of a VSR are composed of the same material.

#### 2.1.2 Controller

During the simulation, voxels vary their area in response to a *control signal* imposed by the controller and to the external forces deriving from the contact with other voxels and with the ground. The control signal is a value in f=[−1,1], where −1 corresponds to maximum requested expansion and 1 corresponds to maximum requested contraction. Expansion and contraction are modeled in the simulation as instantaneous changes of the resting length of the springs in the spring-damper systems of the voxel. The change is such that the area of a voxel subjected to f=1 and no external forces is 75% of the area with f=0, and the area with f=−1 is 125% of that with f=0.

The value of the control signal is set for each voxel at each time-step by the VSR *controller*. Different controllers have been used in previous works, ranging from simple ones where the control signal for each voxel depends only on the time (Kriegman et al., 2018), to others where a recurrent neural network processes the VSR sensor readings to produce the control signals (Medvet et al., 2020a). With the aim of evaluating the adaptability also with respect to different forms of controller, in this study we consider two controllers.

In the first and simplest one, that we call *phase* controller, the control signal of the *i*th voxel at time t=kΔt is given by:

$$f_{i}^{\left(k\right)}=\text{sin}\left(2\pi k\text{Δ}t+\phi_{i}\right)$$ (1)

That is, all the voxels are controlled with a sinusoidal signal with the same amplitude and a frequency of 1 Hz, but different phases $\phi_{i}$. An instance of the phase controller for a VSR with *n* voxels is completely described by the vector $\phi \in {\mathbb{R}}^{n}$ of phases.

The second controller, that we call *neural* controller, is based on a fully connected feedforward artificial neural network (NN) and resembles that proposed by Talamini et al. (2019). At each time step, the controller collects the vector $x^{\left(k\right)}\in {\mathbb{R}}^{m}$ of *m* readings from the VSR sensors and feeds it to the NN; instantaneously, the NN outputs a vector $y^{\left(k\right)}\in {\left[-1,1\right]}^{n}$. The control signal of the *i*th voxel at time is the *i*th element of $y^{\left(k\right)}$. Based on the experimental settings of (Talamini et al., 2019), we here worked with a NN with one hidden layer with 65% of the neurons of the input layer and with the hyperbolic tangent as the activation function. An instance of the neural controller for a VSR with *n* voxels and *m* sensors is hence completely described by the vector $\theta \in {\mathbb{R}}^{p}$ of weights of the NN, with p=0.65(m+1)m+0.65mn, where the +1 is the bias.

When using the neural controller, we equipped each voxel of the VSR with four sensors, resulting in an overall number of sensors m=4n. At each time step, they sense 1) the ratio between the current area of the voxel and its rest area, 2) whether the voxel is or is not touching the ground (the output being 1 or 0, respectively), 3) and 4) the velocity of the center of mass of the voxel along the *x*- and *y*-axes integral with the voxel.

### 2.2 Adaptability

One of the main challenges and opportunities of VSRs is their potential adaptability. Robots made of soft materials may interact with their environment in a more natural way, inspired by how biological systems interact with their environments (Trimmer, 2013).

According to Rus and Tolley (2015), these robotic systems are more adaptable and versatile than traditional ones made of rigid joints. However, this aspect has never been completely explored, since most of the research in the literature has dealt with the automatic design of robots for a very specific task. Kriegman et al. (2018) and Cheney et al. (2013) considered different aspects of soft robots such as the lifetime development and an effective representation and the results are evaluated on their locomotion performance. Sadeghi et al. (2017) engineered a soft robot inspired by the plants for growing. Finally, Shen et al. (2017) took inspiration from cephalopod molluscs for developing a robot that exhibited interesting underwater propulsion and maneuvering mechanisms.

Auerbach and Bongard (2014) showed that the morphological complexity is a result of the selection pressure provided by increased environmental complexity. Recently, Miras et al. (2020) wrote about (Auerbach and Bongard, 2014) that: The authors demonstrated that increasing the complexity of the environmental conditions might result in an increase to the morphological complexity of the creatures. However, measuring complexity does not provide clear insights concerning properties of intelligible morphological traits, for instance, the number of limbs a robot has. Importantly, two environments could be equally complex, but induce the emergence of different phenotypic and behavioral traits. Chvykov et al. (2021) attempted self-organization away from equilibrium through the use of shape-changing robotic active matter, and outlined a methodology for controlling collective behavior. Corucci et al. (2018) investigated the evolution of walking and swimming soft robots in different environments to explore the effect of major environmental transitions during evolution. Horibe et al. (2021) focused on the regeneration of soft robot bodies using growing neural cellular automata when damages are induced to the morphology.

An approach for task-agnostic morphology evolution (TAME) is presented by Hejna et al. (2021), where an information theoretic objective is used instead of the robot performance in a specific task. While the used morphologies are not voxel-based (as in our work) but are encoded as a tree of limbs and joints, the underlying assumption is similar to ours in that morphologies that can achieve a large amount of states in a controllable way may perform well in different tasks. Their approach is based on a graph neural network as predictor of the actions taken by each joint and mutual information-based fitness to maximize the number of possible actions.

Very recently, Bhovad and Li (2021) have proposed soft robots with origami capabilities, which result in a dynamical and high-dimensional system that can be harnessed as physical substrate for RC. The framework of RC in the context of soft robots may provide a novel view on soft robot bodies as dynamical systems and foster the investigation about dynamical behaviors that may be controllable and adaptive.

### 2.3 Reservoir Computing

Reservoir Computing (RC) is a computational framework derived from echo state networks (Jaeger, 2002) and liquid state machines (Fernando and Sojakka, 2003), which leverages on the high-dimensionality of a dynamical system, i.e., its substrate, to produce training-efficient linear readout mechanisms as an alternative to expensive recurrent neural network training. In practice, dynamical systems that possess the echo state property (previous inputs “echo” through the network for a certain amount of time and thereby slowly disappear without being amplified) may be used.

Recent works on RC, such as (Li et al., 2012; Nakajima et al., 2013; Tanaka et al., 2019), suggest that soft robotic systems, thanks to the intrinsically high-dimensionality, non-linearity, and elasticity property of soft materials, which lead to overall highly complex and time-varying dynamics under actuation, are perticularly well suited substrates for RC. Specifically, Li et al. (2012) and Nakajima et al. (2013) showed that the structure of an octopus arm inspired soft robot can be exploited as a computational resource.

Indeed, it has been shown that the rich dynamics used by reservoir computers are often present in systems that show complex behaviors, typically at a critical point between ordered and chaotic dynamics (Schrauwen et al., 2007).

### 2.4 Criticality

The brain criticality hypothesis states that the brain may operate in critical state between ordered and disordered dynamics (Wilting and Priesemann, 2019). Criticality is considered one of the mechanisms by which complexity arises in nature, suggesting the possibility that criticality has been selected by evolution as a beneficial trait (Hesse and Gross, 2014). Theoretical and experimental work has shown that systems at criticality allow for optimal information processing and computing properties (Zimmern, 2020). In practice, in order to achieve criticality, a system parameter has to be precisely tuned. However, some dynamical systems such as the brain have the ability to self-tune through local processes, i.e., self-organized criticality (SOC). SOC is a property typically observed in slowly driven non-equilibrium systems with many degrees of freedom and strongly non-linear dynamics (Bak et al., 1988), which naturally evolve toward a critical point of phase transition between chaotic and non-chaotic regimes (Bertschinger and Natschläger, 2004).

Being close to this phase transition allows systems in which complex computation is possible, as presented by Langton (1990), who investigated the conditions that allow computation in Cellular Automata (CA). Criticality is also inherently connected to the separation property, used by Gibbons (2010) for the assessment of neural reservoirs. Brodeur and Rouat (2012) and Heiney et al. (2019) demonstrated, for spiking neural networks, that the regulation of a RC substrate toward criticality may allow to obtain a more powerful reservoir.

**FIGURE 2:**
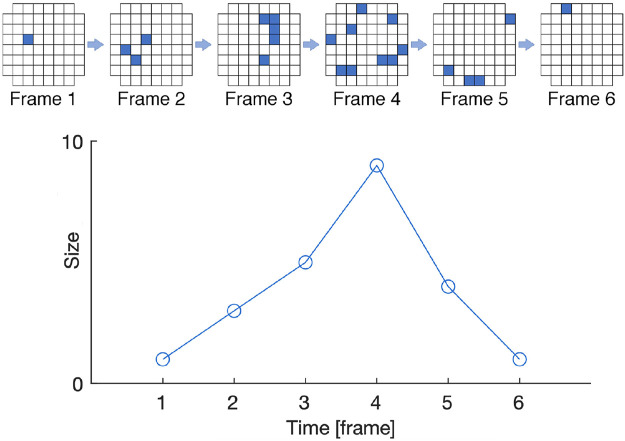
Graphical representation of avalanches in a system composed by 60 cells arranged in a grid. The top row shows an initial activity from a single cell at Frame 1 that propagates to three cells at Frame 2, eventually ending at Frame 6. The bottom row shows the avalanche size and duration. Image adapted from (Heiney et al., 2021) in the context of biological neural networks on Micro-Electrode Arrays.

A standard metric for estimation of criticality is to measure how close is the empirical avalanches distribution in the system (Bak et al., 1988) to a target power law distribution. Such metric exploits a convenient representation for the distributions based on the method presented by Clauset et al. (2009), which is also used in this work. A graphical explanation of avalanche quantification is depicted in Figure 2. An example of using such criticality metric as fitness function in EC is provided in (Pontes-Filho et al., 2020a; Pontes-Filho et al., 2020b), where the goal was to evolve CAs toward SOC.

## 3 Optimizing Voxel-Based Soft Robot Morphology for Criticality

The central hypothesis of this study is that a VSR morphology that exhibits the property of *criticality* is adaptable; that is, VSRs with that morphology, but possibly different controllers, are good in a diverse set of tasks. As a consequence, we speculate that optimizing for criticality results in optimizing for adaptability. In order to verify this hypothesis, we need a way for measuring the degree to which a VSR morphology exhibits the criticality property and a way for optimizing a VSR for criticality. In the following sections, we describe in detail our approaches for both things.

### 3.1 Criticality of a Voxel-Based Soft Robot Morphology

We build our definition of criticality of a VSR morphology based on the concept of *avalanche*. Intuitively, given a dynamical system in which an initial input stimulus can be injected, the avalanche is the resulting outcome and its extension can be measured in time (how long it lasts) and space (how broadly it involves the system). A system that is chaotic will often produce extended avalanches, regardless of the initial input stimulus. Conversely, a system that is predictable and prone to reach equilibrium will in general produce large avalanches only for few input stimuli. Based on this intuitive observation, Bak et al. (1988) showed that systems that lie on the boundary between chaotic and non-chaotic regimes (the *edge of chaos*), i.e., that exhibit criticality, produce avalanches whose extension distribution resembles the power law distribution. Summarizing, the closer the avalanche extension distribution to the power law distribution, the more the system exhibits criticality.

In order to apply this intuitive definition of criticality to the morphology of a VSR, we need to define what is an input stimulus and how to measure the avalanche extension, provided that the morphology itself, i.e., a 2-D grid of voxels, constitutes the dynamical system.

We define as input stimulus the application of a control signal $f_{\text{stimulus}}$ for exactly one time step to exactly one voxel of the morphology. For measuring the avalanche extension, we proceed as follows. We simulate the VSR for $t_{\text{avalanche}}$ simulated time, applying the stimulus at the first time step. Then, at each time step *k* and for each *i*th voxel, we measure the relative area variation:

$$\Delta a_{i}^{\left(k\right)}=\left|\frac{a_{i}^{\left(k\right)}-a_{i}^{\left(k-1\right)}}{a_{0}}\right|$$ (2)

where $a_{i}^{\left(k\right)}$ is the area of the *i*th voxel at *k* and $a_{0}$ is the rest area, the same for every voxel. Finally, we measure the avalanche extension *e* as the number of voxel for which ${\text{max}}_{k}\text{Δ}a_{i}^{\left(k\right)}\geq \tau$, i.e., for which $a_{i}^{\left(k\right)}\geq \tau$ for at least one time step during the simulation—τ is a parameter determining the threshold on the relative area variation. We hence consider only the spatial extension of the avalanche and measure it as the number of voxels which were affected by the initial stimulus.

In order to minimize the impact of the effect of the gravity and to make our definition of criticality agnostic with respect to the morphology rotation, we perform the simulation in absence of gravity.

Based on the above definition of avalanche extension and on the previous studies on avalanche distribution in systems exhibiting criticality, we measure the criticality as follows. Given a morphology *b* composed of *n* voxels, we obtain *n* measures of avalanche extensions $E=\left\{e_{1},\ldots ,e_{n}\right\}$ by applying the initial stimulus once for each of the *n* voxels in *b*—we recall that our simulations are deterministic. Then, we estimate the degree to which the sample *E* fits a power law distribution by computing its coefficient of determination (Nagelkerke et al., 1991) $f_{\text{det}}\left(E\right)\in \left[0,1\right]$ and its Kolmogorov-Smirnov statistic (Drew et al., 2000) $f_{\text{KS}}\left(E\right)\in {\mathbb{R}}^{+}$ computed on *E* and a theoretical power law distribution. Finally, we define the criticality of *b* as:

$$crit\left(b\right)={\left(e^{-f_{KS}\left(E\right)}\right)}^{2}+f_{\det}\left(E\right)$$ (3)

with crit(b)∈[0,2]. The greater its value, the closer the distribution of *E* to the power law distribution, and hence the closer *b* to the edge of chaos.

#### 3.1.1 Choice of the Threshold τ for the Relative Area Variation

In practice, a key role in the quality of the measure of criticality is played by the parameters $f_{\text{stimulus}}$, $t_{\text{avalanche}}$, and τ. Since the mechanical model of the VSR in our simulation employs a damping factor for linear and angular velocities (see Medvet et al. (2020b)), it follows that $t_{\text{avalanche}}$ can be set to a long-enough value: after preliminary experiments, we chose $t_{\text{avalanche}}=30\;\text{s}$—for all the other parameters, including the time step duration of Δt=1/60 s, we used the default values of 2D-VSR-Sim.

Concerning $f_{\text{stimulus}}$ and τ, they obviously interact: the stronger the stimulus, the smaller the threshold needed to count a voxel as contributing to the avalanche. We hence set the largest value for the stimulus, i.e., $f_{\text{stimulus}}=1$, and devised an experimental procedure for finding an appropriate value for τ. Ideally, the threshold value should allow for a large variation in the extension of the avalanches: that is, for a given morphology it should be large enough to have some stimulus for which the avalanche is not widely extended and small enough to have some stimulus for which the avalanche is extended. However, the size of the morphology clearly impacts on such ideal threshold value. For this reason, we assume that τ depends on the number *n* of voxels in the morphology *b* for which the criticality is measured (by means of Eqs. 2, 3) and we fit experimentally a model for τ(n), as follows.

We consider the square morphologies of ℓ×ℓ voxels, with ℓ∈{3,…,10} and, for each one, we: 1) experimentally find the greatest value $\tau_{\text{all}}^{\ell }$ such that every stimulus corresponds to an avalanche of all the $\ell^{2}$ voxels—that is, $\forall \tau >\tau_{\text{all}}^{\ell }$ there is at least one stimulus, i.e., at least one voxel of the morphology to which to apply the initial control signal, that corresponds to an avalanche not involving the entire morphology; 2) experimentally find the lowest value $\tau_{\text{one}}^{\ell }$ such that every stimulus corresponds to an avalanche of only one voxel (the one to which the stimulus is applied).

Then, we consider 10 values for τ evenly distributed in $\left[\tau_{\text{all}}^{\ell },\tau_{\text{one}}^{\ell }\right]$ and, for each one, we measure the standard deviation $\sigma_{E}$ of the corresponding avalanche extensions. Finally, we determine the value $\tau_{\text{opt}}^{\ell }$ that corresponds to the largest $\sigma_{E}$.

This way, we obtain for each one of 10 robots of sizes ranging from n=9 to n=100 an estimate of an optimal threshold value, i.e., a τ value that maximizes the variability of the observed avalanche extensions. We finally determine τ(n) by fitting a quadratic model with those estimates:

$$\tau \left(n\right)=9.24\times 10^{-7}n^{2}-1.42\times 10^{-4}n+6.10\times 10^{-3}$$ (4)

**FIGURE 3:**
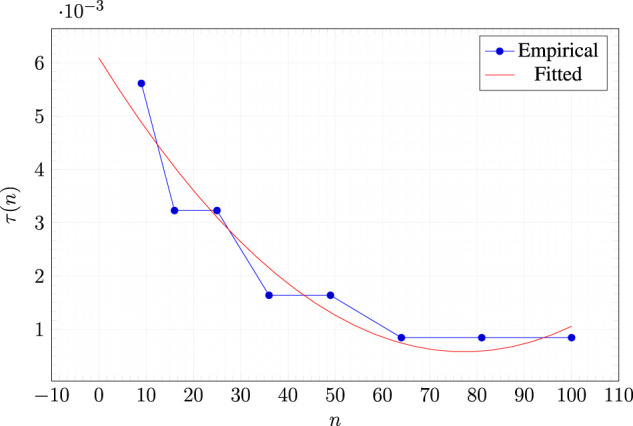
Experimental values for the optimal threshold τ(n) (see text) and corresponding fitted model.

Figure 3 shows the fitted model and the values for τ(n) measured with the procedure described above.

### 3.2 Evolutionary Optimization of Criticality

We aim at optimizing a VSR morphology for criticality, i.e., we want to search in the space of VSR morphologies for the one that corresponds to the largest criticality, as defined in Eq. 3.

We tackle this optimization problem with EC: this large family of optimization methods has been showed to be effective for uncommon search spaces (here, the space of VSR morphologies) and objective functions that are not well characterized. Indeed, EC has been used extensively for the optimization of VSRs: e.g., the morphology in (Cheney et al., 2013), the controller in (Talamini et al., 2019), the sensory apparatus in (Ferigo et al., 2021).

Two key components of any optimization performed by means of EC are the solution representation and the Evolutionary Algorithm (EA). Concerning the former, we represent VSR morphologies as numerical vectors in ${\mathbb{R}}^{\ell^{2}}$. Given a *genotype* $g\in {\mathbb{R}}^{\ell^{2}}$, where ℓ is the side length of a square ℓ×ℓ grid enclosing the largest representable morphology, we obtain the corresponding morphology *b* with the iterative procedure of Algorithm 1. We associate each element of g with a position of an empty ℓ×ℓ grid and iteratively fill the grid with $n_{\text{voxel}}$ voxels, a parameter of the representation, starting from the grid cells corresponding with the largest elements of g and taking care to build a “connected” morphology, i.e., one in which each voxel is reachable from other voxel by moving through adjacent voxels.

Algorithm 1 **:** The algorithm for mapping a genotype $g\in {\mathbb{R}}^{\ell^{2}}$ to a morphology *b* of $n_{\text{voxel}}$ voxels enclosed in a ℓ×ℓ grid.

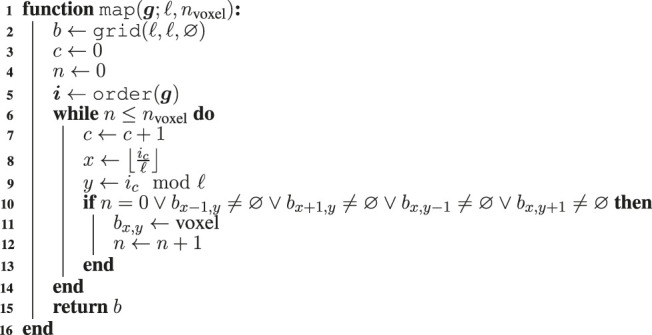

For the evolutionary optimization of the morphology, we use the simple EA of Algorithm 2. We iteratively evolve a population of $n_{\text{pop}}$ solutions for $n_{\text{gen}}$ generations. At each generation, we build the offspring of $n_{\text{pop}}$ individuals by selecting parents with tournament selection and applying mutation or crossover with $p_{\text{mut}}$ or $1-p_{\text{mut}}$ probability. Finally, we merge the offspring and the parents and we discard the worst solutions until the resulting size is again $n_{\text{pop}}$.

Algorithm 2 **:** The EA for the optimization of the morphology.

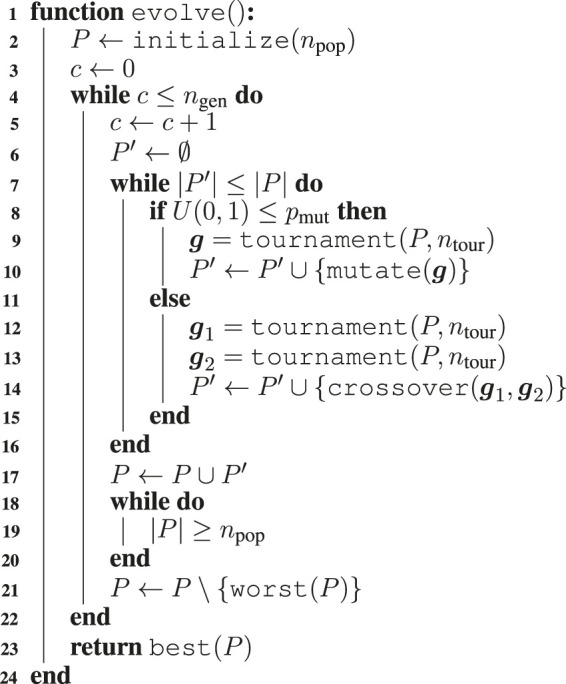

We initialize the population by randomly sampling U(0,1) for each element of each genotype. We select solutions to reproduce with a tournament of size $n_{\text{tour}}$, i.e., we first pick $n_{\text{tour}}$ solutions randomly with repetition, then we select the best one among them. For the mutation, we use the Gaussian mutation, according to which each element ${g^{\text{′}}}_{i}$ of the child $g^{\text{′}}$ is obtained by adding a Gaussian noise with zero mean to the corresponding element $g_{i}$ of the parent g, i.e., ${g^{\text{′}}}_{i}=g_{i}+N\left(0,\sigma_{\text{mut}}\right)$. For the crossover, we use the standard uniform crossover, in which each element ${g^{\text{′}}}_{i}$ of the child $g^{\text{′}}$ is the corresponding element of one of the two parents with uniform probability.

 We remark that we do not optimize the morphology of the VSR for a specific task. Instead, we optimize for criticality, on the assumption that great criticality corresponds to a morphology that is adaptable to different tasks.

## 4 Experiments and Results

In this section, we describe the experimental work carried out to verify our hypotheses. First, we investigate whether it is possible to evolve VSR morphologies that are optimized for criticality, and therefore task-agnostic. In order to quantify the level of criticality of VSR morphologies, we measure the avalanche distribution produced by each morphology as described in Section 3.1.

Second, we test the criticality-evolved morphologies on three different tasks. The research hypothesis here is that the morphologies evolved for criticality perform reasonably well on all tasks, therefore showing adaptation. In this step, we also test some handcrafted morphologies that are expected to be very well suited for specific tasks. While the handcrafted morphologies may be better than the criticality-evolved ones on specific tasks, we expect the overall ability to solve different tasks to be inferior than the criticality-evolved morphologies. In order to test the criticality-evolved morphologies as well as the handcrafted morphologies on the different tasks, we optimize the phase controller (see Section 2.1.2) of several VSRs for each one of the morphologies. We do the optimization with a state-of-the-art EA, detailed below.

Third, we explore whether there are cheaper ways to optimize task-agnostic morphologies with similar dynamical properties as those evolved for criticality. To do that, we start with the observation that VSRs that show higher degree of criticality seem to have morphologies that are less structured, with some degree of randomness in the components stretching out of the bodies. We introduce a pseudo-random generation method that, starting from a voxel, adds a new voxel in one of the neighboring positions. Also, we introduce a random method that adds voxels in random locations until a connected component of a target size is achieved. We measure the criticality of both randomized methods and we test the resulting VSRs on the same three tasks used in the previous experiments (for comparison) by optimizing their phase controllers.

Finally, we investigate whether the criticality-evolved task-agnostic morphologies retain their adaptability also when coupled with a neural controller (see Section 2.1.2), rather than a phase controller. Again, we optimize the controllers for each task and each morphology using a state-of-the-art EA.

We performed all the experiments using publicly available software tools: JGEA^1^ for the evolutionary optimization and 2D-VSR-Sim^2^ for the VSR simulations.

### 4.1 Task-Agnostic Evolution of Voxel-Based Soft Robots Morphology

In this first experiment, we aim at evolving VSR morphologies using a fitness function that is purely based on the criticality measure introduced in Section 3.1. Therefore, the aim is to evolve robot morphologies that are agnostic to the task they may be tested for. Our assumption is that greater criticality corresponds to a morphology that is adaptable, once associated with a controller, to different tasks.

We executed the EA of Section 3.2 ten times with different random seeds. We set the parameters related to the representation to ℓ=10 and $n_{\text{voxel}}=20$: this means that we optimized morphologies of 20 voxels enclosed in a 10×10 grid. Concerning the EA, we used the following parameters: $n_{\text{pop}}=1000$, $n_{\text{gen}}=200$, $\sigma_{\text{mut}}=0.01$, $n_{\text{tour}}=10$. We chose these values after a few preliminary experiments; we verified that small variations did not lead to drastically different results. We obtained one morphology at the end of each execution, i.e., out of each evolutionary *run*. Figure LABEL:fig:fitness-evolution,fig:bodies-criticality-evo summarize the results.

**FIGURE 4:**
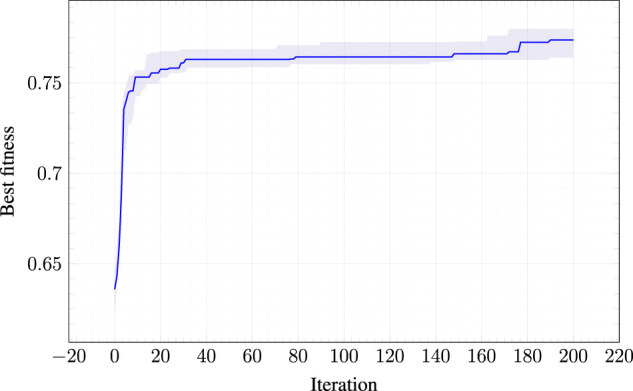
Criticality of the best morphology at each iteration of the EA used for optimizing the morphologies. The solid lines indicates the median across the 10 runs; the shaded area corresponds to the interquartile range (from 25-th to 75-percentile).

Figure 4 shows the criticality of the best morphology during the evolution (median value and interquartile range across the 10 runs). It can be seen that there is an improvement of the criticality: the largest portion of the improvement is obtained in the first stage of the evolution. The figure also shows that there is still an intrinsic boundary which prevents the evolution to find a morphology with maximum theoretical criticality, i.e., crit(b)=2—see Eq. 3. We speculate that larger values for the criticality could be obtained with different, possibly larger, values for $n_{\text{voxel}}$, i.e., with morphologies with more voxels. However, what matters is that the morphologies obtained from the evolutionary process have reached higher criticality values than all the other baselines presented in the following experiments.

**FIGURE 5:**
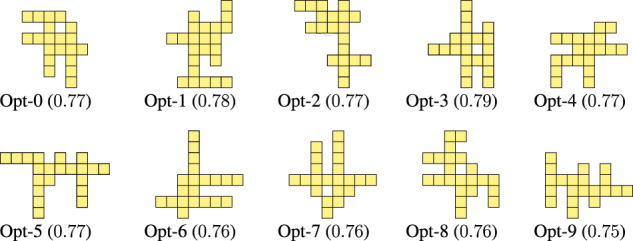
Morphologies evolved for criticality together with the corresponding value of criticality.

Figure 5 shows the final 10 optimized morphologies, i.e., for each run, the morphology of the final population with the largest value for the criticality. The evolved morphologies have several offshoots that are stretching out from the “central core” (i.e., the central portion of the morphology). Apparently, there is not a recurrent pattern in the placement, orientation, and length of those offshoots. In addition, the largest central cores are fairly small, ranging from rectangles of size 2×2 to 2×3, and only a single case with a rectangle of size 3×3 voxels. This is in stark contrast with morphologies traditionally tested in the literature, which typically involve highly structured and compact bodies.

### 4.2 Validating the Adaptation to Different Tasks

In order to test how adaptable the task-agnostic morphologies evolved in the previous experiment are, we consider three *tasks*. For each task and each morphology, we evolve a phase controller of a VSR with that morphology; we drive the evolution with a measure of how well the VSR is performing the task. The more adaptable a task-agnostic morphology, the better the performance on all tasks of VSRs with evolved task-specific controllers and that morphology.

#### 4.2.1 Tasks

We considered the tasks described below. In each task, we simulate the VSR under evaluation for a fixed time in a predefined, task-specific environment (with gravity); upon the simulation, we take a quantitative measure *f* of the degree of *task achievement*.

In the *locomotion* task, the VSR has to run along a flat terrain the farthest possible. The simulation lasts 20 s (simulated time) and we measure the average velocity of the VSR along the positive *x*-axis, i.e., we measure the difference between the *x*-coordinate of the center of mass of the VSR at t=20 s and at t=0 s and divide it by 20.

In the *jump* task, the VSR has to jump the highest possible. The simulation lasts 20 s and we measure the height of the highest jump done in that interval, i.e., the difference between the maximum observed value of the *y*-coordinate of the center of mass and its value at t=0 s—the latter is considered as a reference point to allow a fair comparison of VSRs whose morphology extends mainly along different directions. In this task, the environment is limited by two walls on left and right: interestingly, some of the VSRs evolved to attempt to climb those walls, rather than to actually jump.

**FIGURE 6:**
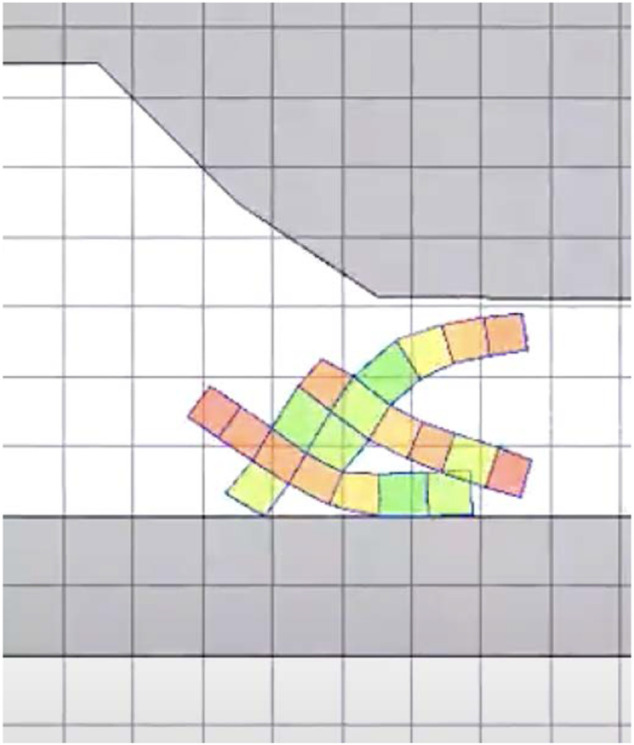
Example of cave-like environment used in the escape task.

Finally, in the *escape* task, the VSR has to exit from a cave-like environment. Here the VSR initially starts in an environment with a ceiling, a wall on the left, and a narrow aperture on the right. An example of a cave-like environment is shown in Figure 6, where the aperture has an initial height of 75% of the VSR largest dimension, and decreases slowly. The simulation lasts 40% and we measure the distance “walked” by the VSR along the positive *x*-axis, as for the locomotion task. Differently from the latter, however, the VSR here does not only need to exhibit a gait that allows to move effectively, but it also needs to “squeeze” itself in order to actually pass through the aperture.

#### 4.2.2 Controller Evolution

Given a morphology and a task, we evolve a phase controller using Covariance Matrix Adaptation Evolutionary Strategies (CMA-ES) (Hansen et al., 2003). CMA-ES is widely considered as a state-of-the-art EA for continuous optimization, i.e., for optimization in the space ${\mathbb{R}}^{p}$. Since a phase controller is defined by the numerical vector of the *n* phases (see Section 2.1.2), *n* being the number of voxels in the morphology, CMA-ES perfectly fits the case. Indeed, it has been recently shown that this EA works well also for VSRs (Ferigo et al., 2021; Medvet and Bartoli, 2021).

CMA-ES iteratively optimizes a numerical vector in the form of a multivariate normal distribution. At each iteration, it samples the distribution obtaining a number λ of solutions and then updates the parameters of the distribution by recomputing them based on the best $\lfloor \frac{\lambda }{2}\rfloor$ solutions, i.e., those with the largest task achievement measure *f*. In the latter operation, CMA-ES employs non trivial heuristics that are detailed in (Hansen, 2016).

We used CMA-ES with the default parameters suggested in (Hansen, 2016): we set the initial step size σ=0.5 and the population size λ=4+⌊3⁡log⁡p⌋, $p=n_{\text{voxel}}=20$ being the dimension of the search space. We set the initial vector of means of the multivariate normal distribution by sampling uniformly the interval [−1.0,1.0] for each vector element. We stopped the execution of CMA-ES after 10,000 fitness evaluations.

#### 4.2.3 Comparison Baselines: Handcrafted Morphologies

**FIGURE 7:**
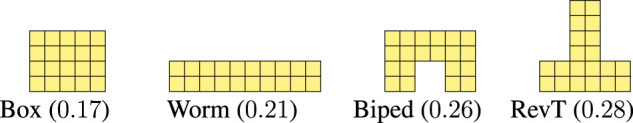
Handcrafted morphologies, designed based on domain knowledge, together with the corresponding value of criticality.

To benchmark the adaptability of the criticality-evolved morphologies, we consider also four manually designed morphologies that we show in Figure 7.

For the *Worm* and the *Biped* morphologies, we got inspiration by the results of (Talamini et al., 2019; Medvet et al., 2020b; Medvet et al., 2020c), where they have been thoroughly assessed in the task of locomotion. The *Box* and *RevT* are some of the simplest morphologies that can be manually designed. All these morphologies are made of exactly $n_{\text{voxel}}=20$, and they all exhibit a low criticality score.

#### 4.2.4 Results and Discussion

**FIGURE 8:**
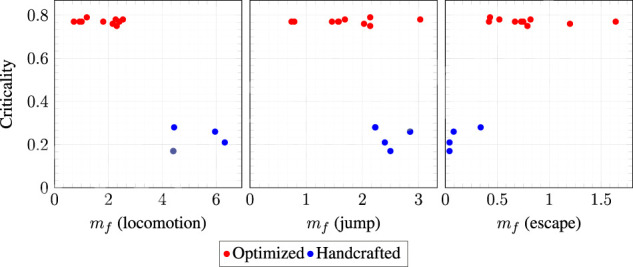
Scatter plots of the criticality vs. the median task achievement degree $m_{f}$, one point for each morphology, one plot for each task.

For each task and each morphology, we executed CMA-ES 10 times with different random seeds. We hence performed 3×(10+4)×10 evolutionary runs. After each run, we took note of the task achievement degree *f* of the best final VSR. Then, for each morphology and task, we computed the median $m_{f}$ and the standard deviation $\sigma_{f}$ of *f* across all the 10 runs. Once we completed all the runs for a task, we ranked the morphologies according to their $m_{f}$ on that task and assigned a task-specific rank *r* to each morphology. Finally, we computed for each morphology the average rank $\mu_{r}$ by considering the mean of the ranks on the three tasks: the lower $\mu_{r}$, the greater the adaptability. Table 1 and Figure 8 summarize the results.

**TABLE 1:** Results for the optimized and handcrafted morphologies coupled with the phase controller. For each task and each morphology, the table shows the median $m_{f}$ and the standard deviation $\sigma_{f}$ of *f* across all the 10 runs. Moreover, the table also shows the task-wize ranks *r*, the average rank $\mu_{r}$, and the criticality of each morphology.

	Locomotion			Jump			Escape				
Morph	$m_{f}$	$\sigma_{f}$	*r*	$m_{f}$	$\sigma_{f}$	*r*	$m_{f}$	$\sigma_{f}$	*r*	Crit	$\mu_{r}$
Opt-1	2.54	0.2	5	3.03	0.3	1	0.82	0.3	3	0.78	3
Biped	5.96	1.3	2	2.85	0.3	2	0.08	0.0	12	0.26	5
Opt-9	2.31	0.7	7	2.14	0.4	6	0.79	0.1	4	0.75	6
Opt-6	2.16	0.5	9	2.03	0.4	8	1.20	0.3	2	0.76	6
RevT	4.44	5.2	3	2.23	0.2	5	0.34	0.0	11	0.28	6
Worm	6.32	0.6	1	2.40	0.6	4	0.04	0.0	14	0.21	6
Box	4.41	1.0	4	2.50	0.4	3	0.04	0.0	13	0.17	7
Opt-2	1.81	0.5	10	0.79	0.0	13	1.64	0.4	1	0.77	8
Opt-5	2.27	0.5	8	1.69	0.3	9	0.52	0.2	8	0.78	8
Opt-3	1.20	0.2	11	2.14	0.3	7	0.43	0.2	9	0.79	9
Opt-4	2.41	0.6	6	1.57	0.3	11	0.42	0.2	10	0.77	9
Opt-0	0.93	0.4	13	1.58	0.2	10	0.73	0.1	6	0.77	10
Opt-7	1.01	0.4	12	1.46	0.1	12	0.67	0.1	7	0.77	10
Opt-8	0.72	0.1	14	0.74	0.4	14	0.75	0.1	5	0.77	11

Table 1 shows all the salient indices for the 10 optimized morphologies (denoted by Opt-*n*, with n∈{0,…,9}) and the four handcrafted morphologies. The morphologies are sorted according to their average rank $\mu_{r}$. The foremost finding is that the morphology that ranks, on average, the best on the three tasks is an optimized morphology, namely Opt-1. That is, Opt-1 is the most adaptable morphology among the 14 considered morphologies.

A more detailed analysis of the results can be done by observing the rankings task by task. Considering only the results on the locomotion task, it is clear that the handcrafted morphologies, namely Worm, Biped, RevT, and Box, are actually the most effective ones. However, it can also be seen from Table 1 that some of the handcrafted morphologies, while being often extremely effective on a specific task, perform poorly on other tasks. For instance, the Worm is the most effective in locomotion and the least effective in escape.

The above considerations are corroborated by Figure 8. The figure shows, for each task, a scatter plot of the median task achievement degree $m_{f}$ vs. the criticality, one point for each morphology. It can be seen that the handcrafted morphologies are very effective in the locomotion task (in facts, two of them have been designed and extensively used purposely for that task) while they are all poorly effective for the escape task. Interestingly, the only handcrafted morphology that performs decently on the escape task (RevT) is also the one with the largest criticality.

We performed an analysis of the statistical significance of the results of this experiment. Precisely, we aimed at testing two null hypotheses. First, we tested, for each task, the null hypothesis that morphologies optimized for criticality obtain the same median task achievement degree $m_{f}$ of handcrafted morphologies. We verified that the null hypothesis can be rejected for two on three tasks, since the difference between the corresponding $m_{f}$ values is statistically significant (Mann-Whitney *U* test with α=0.05 and Bonferroni correction: *p*-value is $0.002\leq \frac{\alpha }{m}$, $0.028>\frac{\alpha }{m}$, and $0.006\leq \frac{\alpha }{m}$, with m=3, respectively for locomotion, jump, and escape). Second, we tested the null hypothesis that, considering all tasks, morphologies optimized for criticality achieve the same overall average rank $\mu_{r}$ of handcrafted morphologies. We verified that the null hypothesis cannot be rejected, since the difference of $\mu_{r}$ values is not statistically significant (*p*-value is 0.14>α).

### 4.3 A Cheap Proxy for Task-Agnostic Evolution

In the previous experiments, we observed that the criticality appears to be a good predictor of the adaptability: VSRs based on morphologies evolved for criticality using a task-agnostic optimization showed to be good, on average, on different tasks. However, evolution toward criticality is a resources intensive process. We hence explored the possibility of designing a generative procedure able to build, from scratch and in a one-shot fashion, a morphology that exhibits good criticality.

For designing that procedure, we observed the features of the optimized morphologies (see Figure 5). We also considered the raw results of the experiments of Section 4.1. The solutions in the initial populations of the runs of the criticality optimizations exhibited criticality values larger than those of the handcrafted morphologies (see Figure 4): that is, the joint result of the genotype initialization and the solution representation produced alone, without evolutionary pressure, morphologies with larger criticality than the handcrafted ones. Based on these considerations, we devised a non-deterministic procedure for generating morphologies with good criticality, as follows.

**FIGURE 9:**
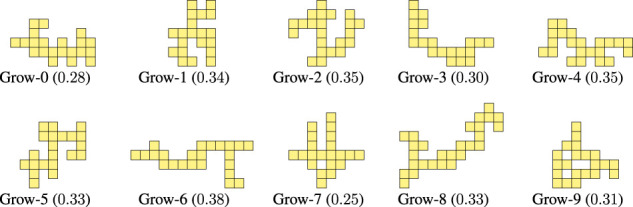
Morphologies generated with the Grow procedure together with the corresponding value of criticality.

Given an unlimited empty grid, we start by putting a voxel in a randomly chosen position in the grid. Then, we iteratively add one voxel at a position adjacent to the last added voxel until $n_{\text{voxel}}$ voxels have been added. By adding each voxel close to the last one, the overall morphology ends up having those kind of offshoots that we observed in the optimized morphologies. We call this procedure *Grow*. We executed it 10 times with $n_{\text{voxel}}=20$ and different random seeds: Figure 9 shows the 10 obtained morphologies along with their criticality value.

**FIGURE 10:**
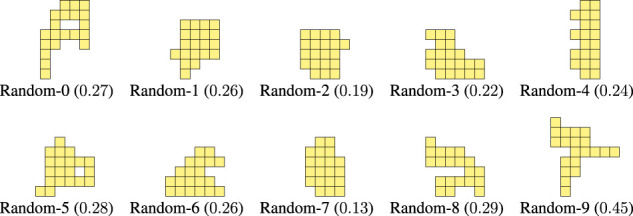
Morphologies generated with the Random procedure together with the corresponding value of criticality.

As a comparison baseline, we also devised another non-deterministic procedure that still generates morphologies randomly, but does not attempt to mimic the shape of the optimized morphologies, namely their offshoots. In this case, we simply add voxels at random positions of an initially empty ℓ×ℓ grid until we obtain a connected portion of the grid consisting of $n_{\text{voxel}}$ voxels. We call this procedure *Random*. As for the Grow procedure, we executed the Random procedure 10 times with different random seeds—and with ℓ=10 and $n_{\text{voxel}}=20$: Figure 10 shows the 10 obtained morphologies along with their criticality value.

By looking at Figure LABEL:fig:bodies-grow,fig:bodies-random it can be seen that the Grow morphologies are visually more similar to the optimized ones (see Figure 5) than the Random morphologies. Moreover, the criticality of the Grow morphologies is in general larger than the criticality of the Random morphologies. We remark that both procedures are task-agnostic, as the criticality-driven evolution of the morphology.

#### 4.3.1 Adaptability of Grow Morphologies

As a further validation of the fact that the Grow procedure can be a cheap proxy for the criticality-driven evolution of morphologies, we repeated experiments of Section 4.2 for the 10 Grow morphologies of Figure 9. We also included the 10 Random morphologies of Figure 10. We hence performed other 3×(10+10)×10 evolutionary runs of CMA-ES. Table 2 summarizes the results.

**TABLE 2:** Results for the optimized and randomly generated morphologies coupled with the phase controller. For each task and each morphology, the table shows the median $m_{f}$ and the standard deviation $\sigma_{f}$ of *f* across all the 10 runs. Moreover, the table also shows the task-wize ranks *r*, the average rank $\mu_{r}$, and the criticality of each morphology.

Morph	Locomotion			Jump			Escape				
	$m_{f}$	$\sigma_{f}$	*r*	$m_{f}$	$\sigma_{f}$	*r*	$m_{f}$	$\sigma_{f}$	*r*	Crit	$\mu_{r}$
Grow-4	3.02	0.9	1	2.89	0.8	3	1.21	0.2	7	0.35	4
Grow-5	2.86	0.5	4	3.37	1.0	1	1.07	0.3	11	0.34	5
Opt-1	2.54	0.2	5	3.03	0.3	2	0.82	0.3	15	0.78	7
Opt-6	2.16	0.5	10	2.03	0.4	7	1.20	0.3	8	0.76	8
Random-6	2.03	0.2	11	1.58	0.2	14	2.09	0.1	1	0.26	9
Grow-9	2.94	0.3	3	1.98	0.3	9	1.02	0.2	14	0.31	9
Random-5	1.54	0.2	14	1.66	0.2	12	1.66	0.2	2	0.28	9
Opt-9	2.31	0.7	8	2.14	0.4	4	0.79	0.1	16	0.75	9
Grow-0	2.97	0.3	2	2.00	0.5	8	0.69	0.2	22	0.28	11
Random-8	1.35	0.2	18	1.42	0.2	19	1.60	0.2	4	0.29	14
Opt-2	1.81	0.5	13	0.79	0.0	26	1.64	0.4	3	0.77	14
Random-3	1.42	0.2	17	1.32	0.2	21	1.58	0.3	5	0.22	14
Grow-1	1.90	0.3	12	2.06	0.3	6	0.51	0.1	25	0.34	14
Opt-5	2.27	0.5	9	1.69	0.3	11	0.52	0.2	24	0.78	15
Grow-8	2.46	0.4	6	1.62	0.1	13	0.20	0.0	30	0.34	16
Opt-3	1.20	0.2	19	2.14	0.3	5	0.43	0.2	26	0.79	17
Grow-3	0.98	0.4	25	1.39	0.4	20	1.23	0.4	6	0.30	17
Opt-4	2.41	0.6	7	1.57	0.3	16	0.42	0.2	28	0.77	17
Random-0	1.01	0.1	23	1.46	0.2	18	1.07	0.1	12	0.23	18
Random-2	1.01	0.0	24	1.24	0.1	22	1.16	0.1	9	0.19	18
Random-1	1.47	0.1	15	1.12	0.2	24	0.77	0.0	18	0.26	19
Grow-2	1.17	0.4	20	1.71	0.5	10	0.43	0.1	27	0.35	19
Grow-7	1.13	0.1	21	0.77	0.2	28	1.15	0.1	10	0.26	20
Opt-0	0.93	0.4	26	1.58	0.2	15	0.73	0.1	20	0.77	20
Opt-7	1.01	0.4	22	1.46	0.1	17	0.67	0.1	23	0.77	21
Random-4	0.78	0.1	27	0.89	0.0	25	1.03	0.1	13	0.25	22
Random-7	0.65	0.0	29	1.18	0.2	23	0.78	0.0	17	0.13	23
Grow-6	1.46	0.4	16	0.77	0.2	27	0.32	0.2	29	0.38	24
Opt-8	0.72	0.1	28	0.74	0.4	29	0.75	0.1	19	0.77	25
Random-9	0.58	0.0	30	0.54	0.0	30	0.70	0.1	21	0.45	27

It can be seen that the Grow procedure, i.e., the one inspired by the results of the criticality-driven evolution results, generates morphologies that exhibit, on average, a greater adaptability (i.e., lower $\mu_{r}$) than the Random morphologies. Interestingly, the two most adaptable morphologies are Grow-4 and Grow-5, that outperform even the ones evolved toward criticality, in terms of overall ranking.

We performed the same statistical significance analysis of Section 4.2.4. Considering $m_{f}$ separately for each task, the difference between Grow and optimized is never statistically significant and the difference between Grow and handcrafted is statistically significant for two on three tasks (jump not significant). Considering $\mu_{r}$, the differences between optimized morphologies and each one of the other types are not statistically significant.

A few videos of evolved VSRs with the morphologies obtained with the procedures above in the three considered tasks are available online: locomotion (https://www.youtube.com/watch?v=rRPI-WvezY0), jump (https://www.youtube.com/watch?v=5JYwQPptVZE), escape (https://www.youtube.com/watch?v=1G8eO5DCRLM).

### 4.4 Validating the Adaptation to Different Controllers

We further investigated the adaptability of the morphology generated with the task-agnostic procedures described above—namely, the criticality-driven evolution, the Grow, and the Random procedures, by repeating the same validation process of the previous sections with a different controller. Here, we used the neural controller described in Section 2.1.2. This controller is more complex than the phase one for two reasons: 1) it is based on a neural network, and can hence generate a richer dynamics in the control signals and 2) it collects and processes the readings of the sensors the robot is equipped to.

We speculate that the ability to sense itself and the environment might be ever more beneficial with irregular morphologies.

We performed the 3×(10+10+10+4)×10 runs of CMA-ES, whose outcomes we summarize in Table 3—we remark that the dimension of the search space was here much larger than the case of the phase controller (p≈2000 vs. p=20). The table shows, for each morphology, the criticality value and the average rank $\mu_{r}$. We present two views of the results: on the left, we compare optimized and handcrafted morphologies; on the right, we compare the morphologies obtained with the three task-agnostic procedures.

**TABLE 3:** Results for the optimized and randomly generated morphologies coupled with the neural controller. For each morphology, the table shows the average rank $\mu_{r}$ and the criticality of each morphology; $\mu_{r}$ is computed for optimized and handcrafted morphologies, on the left, and for the morphologies obtained with the three task-agnostic procedures, on the right.

Morph	Crit	$\mu_{r}$
Opt-5	0.78	1
Opt-7	0.77	2
Opt-1	0.78	2
Opt-6	0.76	4
Opt-9	0.76	7
Opt-4	0.77	7
Worm	0.22	7
Opt-8	0.77	9
RevT	0.29	9
Biped	0.26	9
Opt-2	0.78	9
Box	0.17	11
Opt-0	0.78	12
Opt-3	0.79	14
Opt-5	0.78	1
Grow-5	0.34	2
Opt-1	0.78	3
Opt-7	0.77	4
Random-0	0.28	4
Random-9	0.46	6
Random-1	0.26	7
Opt-6	0.76	8
Random-5	0.28	10
Opt-9	0.76	12
Grow-2	0.35	12
Opt-4	0.77	12
Grow-4	0.35	14
Random-8	0.30	15
Opt-2	0.78	15
Opt-8	0.77	16
Random-6	0.27	16
Grow-1	0.34	17
Random-2	0.19	18
Random-3	0.22	20
Random-7	0.13	20
Grow-3	0.30	22
Opt-0	0.78	23
Grow-0	0.28	24
Random-4	0.25	24
Opt-3	0.79	26

Looking at the results presented in Table 3, our thesis is further corroborated. On one hand, the number of optimized morphologies that exhibit greater adaptability (i.e., lower $\mu_{r}$) than the handcrafted morphologies is larger than the case of the phase controller. On the other hand, three of the top four morphologies are those evolved for criticality, rather than generated randomly with the Grow or Random procedures. As for the results with the phase controller, the differences in average rank $\mu_{r}$ between optimized morphologies and each one of the other types are not statistically significant. These results suggest that a more complex controller may be needed to exploit the full potential of morphologies evolved for criticality, i.e., for showing a complex behavior between order and chaos.

## 5 Conclusion

In this work, we have introduced a task-agnostic approach for measuring and predicting the adaptability potential of a soft robotic body based on the definition of criticality, a property of dynamical systems that operate at a phase transition between order and disorder. To this extent, we have proposed an algorithm for guiding the automatic design of adaptable soft robotic morphologies by means of evolutionary computation that, without the need to define a specific target task, relies entirely on the resulting dynamics of the robots morphology measured as the avalanche distributions, i.e., how close they are to a power law distribution. The morphologies evolved with our method obtain criticality values far greater than those handcrafted with domain knowledge to perform specific tasks.

We have then validated the evolved morphologies on three tasks requiring different motor skills, against some manually designed morphologies inspired by previous works where domain knowledge is considered, using a simple non-sensing control algorithm based on periodic control signals. The results show that our criticality-based approach results into more adaptable robots, i.e., performing reasonably well in the different tasks, w.r.t. the manually crafted ones. On the other hand, the robots optimized for specific tasks perform poorly on other tasks.

Motivated by the computational cost of the criticality optimization, we have then considered other design algorithms based on randomness, which on the contrary are not computationally expensive. Such randomized algorithms may produce morphologies that are visually similar to those evolved for criticality, but with criticality values that are inferior to the evolved ones (while being higher that the handcrafted robots). In addition, such morphologies generated with randomized methods show sometimes promising adaptability.

Finally, we have repeated the validation process to investigate whether a sensing controller based on neural networks would also be able to control the criticality evolved robots, and thus show adaptability to the different tasks. Neural network sensing controllers allow the robots with optimized morphologies to leverage their body complexity more than non sensing controllers. Provided with distributed sensing, the criticality evolved morphologies reach the highest overall ranking when compared to any baseline. This promising result suggests that morphologies supporting critical behavior are more adaptive independently of the type of controller being used, and independently of the target task.

## Data Availability

The raw data supporting the conclusions of this article will be made available by the authors, without undue reservation.

## Footnotes

1
https://github.com/ericmedvet/jgea.

2
https://github.com/ericmedvet/2dhmsr.
